# In Vitro Effects of Enniatin A on Steroidogenesis and Proliferation of Bovine Granulosa Cells

**DOI:** 10.3390/toxins14100714

**Published:** 2022-10-20

**Authors:** Ilaria Chiminelli, Leon J. Spicer, Excel Rio S. Maylem, Francesca Caloni

**Affiliations:** 1 Department of Environmental Science and Policy (ESP), Università degli Studi di Milano, 20133 Milan, Italy; 2 Department of Animal and Food Sciences, Oklahoma State University, Stillwater, OK 74078, USA

**Keywords:** enniatin A, estradiol, progesterone, granulosa cell, in vitro

## Abstract

The emerging *Fusarium* mycotoxins enniatins (ENNs) have been the focus of new research because of their well-documented existence in various cereal and grain products. Research findings indicate that reproductive disorders may be caused by exposure to *Fusarium* mycotoxins, but little work has evaluated ENNs on reproductive function. Therefore, to determine the effects of ENNA on the proliferation and steroidogenesis of granulosa cells (GC), experiments were conducted using bovine GC cultures. In vitro, ENNA (1–5 μM) inhibited (*p* < 0.05) hormone-induced GC progesterone and estradiol production. The inhibitory effect of ENNA on estradiol production was more pronounced in small- than large-follicle GC. In large-follicle GC, 0.3 μM ENNA had no effect (*p* > 0.10) whereas 1 and 3 μM ENNA inhibited GC proliferation. In small-follicle GC, ENNA (1–5 μM) dramatically decreased (*p* < 0.05) GC proliferation. Using cell number data, the IC_50_ of ENNA was estimated at 2 μM for both follicle sizes. We conclude that ENNA can directly inhibit ovarian function in cattle, decreasing the proliferation and steroid production of GC.

## 1. Introduction

Molds produce several species of mycotoxins [1,2,3] that contaminate various foodstuffs, feedstuffs, and cereals worldwide including wheat, barley, corn, and rice [4,5]. These mycotoxins have acute and chronic toxicity and cause carcinogenic and mutagenic effects as well as reproductive toxicity [4,6,7,8]. Among the emerging mycotoxins, defined as “mycotoxins, which are neither routinely determined, nor legislatively regulated” [9] are the enniatins (ENNs; A, A1, B, B1) which have been isolated in food and feed products and pose a serious risk on human and animal health [10,11,12,13]. Some data exists regarding ENNs toxicity, concentration levels, occurrence, and metabolism [7,8,14,15,16,17,18]. A recent review summarizes the effects of ENNB in vivo as well as its in vitro toxicity in several non-reproductive cell lines [8]. After oral administration of ENNB1 in pigs, fast gastrointestinal absorption occurred [19]. Intravenous administered ENNB1 caused moderate distribution in pigs and chickens [11,19,20]. However, the effects of ENNs, particularly ENNA on reproductive function in farm animals are lacking. Recent studies suggest that ENNs are cytotoxic to embryos [21,22]. Using mouse blastocysts, 5 and 10 μM ENNB1 exerted cytotoxic effects and induced a reduction of inner cell mass cells in blastocysts [22]. In ovarian studies, the effects of other Fusarium mycotoxins (e.g., fumonisin B1 and beauvericin) on granulosa cell (GC) function have shown that they inhibit cell proliferation and steroidogenesis in cultured GC from pigs [23] and cattle [24]. However, the effects of ENNs on ovarian function have not been explored. Therefore, the goal of the present study was to determine the effects of ENNA on reproductive function in cattle, and in particular, if ENNA has direct effects on granulosa cell (GC) proliferation and steroidogenesis. 

## 2. Results

### 2.1. Experiment 1: Inhibitory Effects of ENNA on Large-Follicle GC Numbers and Steroid Production

**Figure 1:**
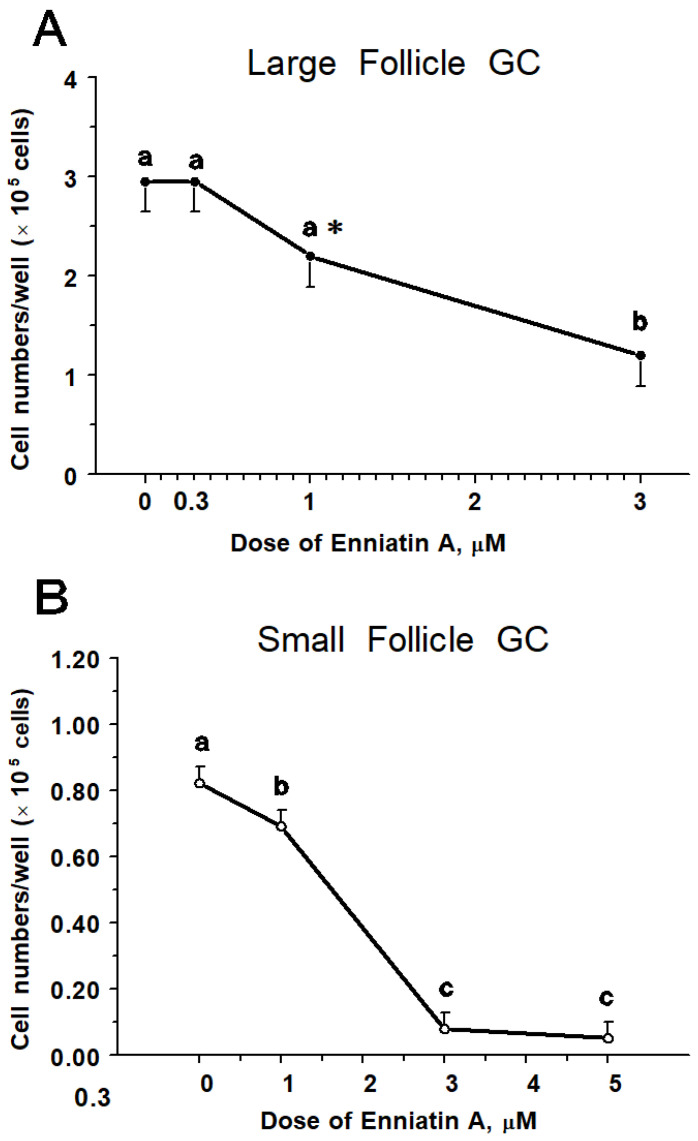
Effect of enniatin A on cell numbers after 48 h exposure at 0, 0.3, 1, and 3 µM on bovine granulosa cells (GC) harvested from large follicles (**A**). Effect of enniatin A on cell numbers after 48 h exposure at 0, 1, 3, and 5 µM on bovine GC harvested from small follicles (**B**). Within a panel, means without a common letter (a–c) differ (*p* < 0.05); asterisk indicates mean tends to differ (*p* < 0.10) from 0 and 0.3 µM means in Panel A.

Numbers of large-follicle GC decreased by 30% (*p* = 0.09) and 60% (*p* < 0.01) when ENNA was applied for 2 days at 1 and 3 µM, respectively (Figure 1A). However, 0.3 µM ENNA had no effect (*p* > 0.10) on GC numbers.

**Figure 2:**
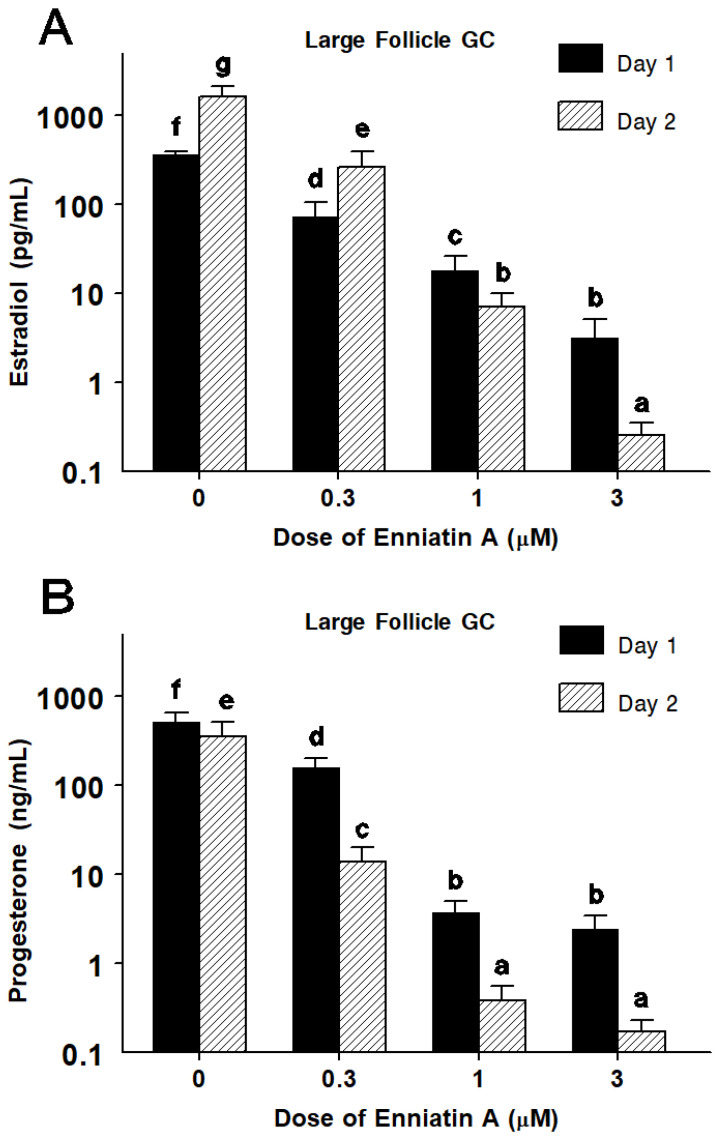
Effect of enniatin A on cell estradiol (**A**) and progesterone (**B**) production after 24 h and 48 h exposure at 0, 0.3, 1, and 3 µM on bovine granulosa cells (GC) harvested from large follicles. Note the log scale for steroid production. Within a panel, means without a common letter (a–g) differ (*p* < 0.05).

In large-follicle GC, the main effect of ENNA was significant causing a dose-dependent decrease (*p <* 0.05) in estradiol (Figure 2A) and progesterone (Figure 2B) production.

After 1 and 2 days of treatment, ENNA at 0.3, 1 and 3 µM inhibited (*p* < 0.0001) estradiol production by large-follicle GC by over 80% (Figure 2A) and progesterone production by over 70% (Figure 2B).

### 2.2. Experiment 2: Inhibitory Effects of ENNA on Small-Follicle GC Numbers and Steroid Production

**Figure 3:**
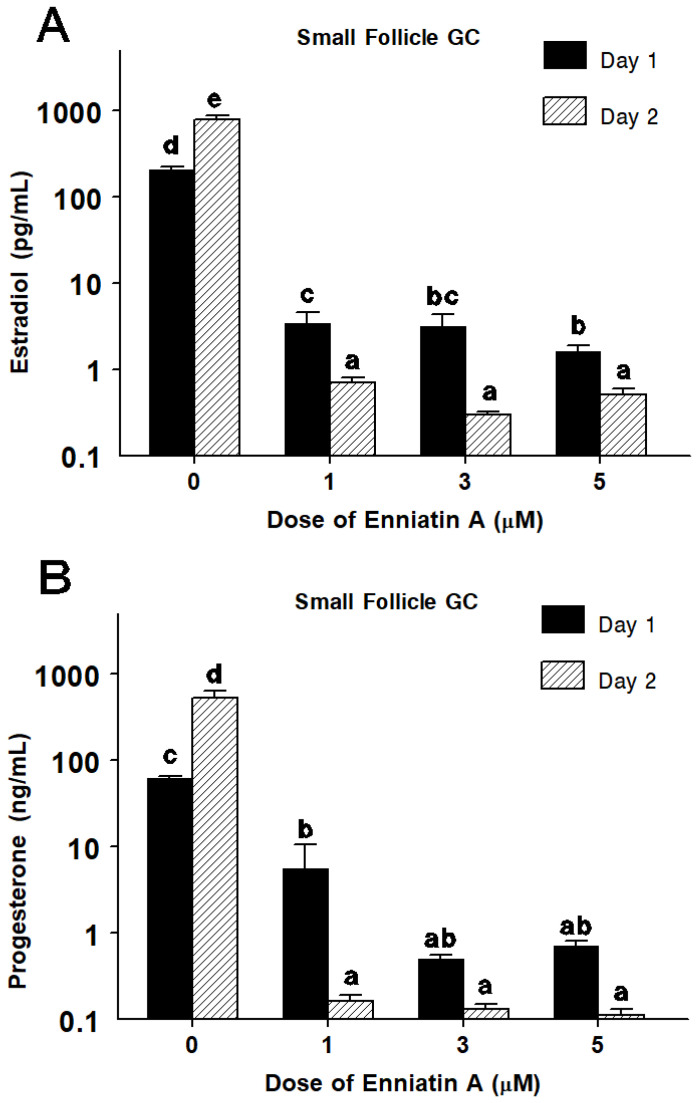
Effect of enniatin A on estradiol (**A**) and progesterone (**B**) production after 24 h and 48 h exposure at 0, 1, 3, and 5 µM on bovine granulosa cells (GC) harvested from small follicles. Note the log scale for steroid production. Within a panel, means without a common letter (a–e) differ (*p* < 0.05).

In small-follicle GC, the main effect of ENNA was significant causing a dose-dependent decrease (*p <* 0.05) in cell proliferation (Figure 1B) and estradiol (Figure 3A) and progesterone (Figure 3B) production.

Specifically, 2-day treatment with ENNA significantly reduced GC numbers by 10, 90, and 95% when applied at 1, 3, and 5 µM, respectively (Figure 1B). ENNA at 1, 3, and 5 µM inhibited (*p* < 0.0001) estradiol production by over 99% after 1-day and 2-day exposure (Figure 3A). Similarly, progesterone production was inhibited (*p* < 0.01) by over 90% after exposure to ENNA at 1, 3 and 5 µM (Figure 3B). Using cell number data, the IC_50_ of ENNA was estimated at 2 μM for both follicle sizes.

## 3. Discussion

Results of the present study indicate that: (1) ENNA inhibited steroid production induced by FSH plus IGF1 in GC and that the inhibitory effect of ENNA on estradiol production was much more pronounced in small- vs. large-follicle GC; and (2) ENNA inhibited proliferation of GC. Thus, the results of the present study indicate for the first time that ENNA may directly inhibit ovarian follicular function in cattle.

The effects of ENNA on steroid production by GC have not been previously reported. However, other Fusarium mycotoxins such as beauvericin [7,24], deoxynivalenol [5,23], and fumonisin B1 [23,24] have been shown to inhibit estradiol and progesterone secretion as well as GC proliferation in porcine and bovine GC [5,7,11,23,24]. Some of these previous studies have suggested direct mycotoxin inhibition of *CYP19A1* and *CYP11A1* mRNA abundance [23,24], and thus should be the focus of future studies with ENNA. Interestingly, 0.3 µM ENNA had no effect on large-follicle GC numbers but significantly reduced both estradiol and progesterone production suggesting that the steroidogenic machinery of GC is more sensitive to ENNA than is the processes of cell proliferation.

Regarding cell proliferation, Fusarium mycotoxins such as ENNB have been reported to disturb the normal cell cycle and thus have anti-proliferative effects on several cell types [25]. For example, Gammelsrud et al. (2012) [26] using the murine monocyte-macrophage RAW 267.4 cell line found that 24 h treatment with ENNB (from 1.25 to 10 µM) caused an increase in the percentage of cells in G0/G1 phase. Similarly, Juan-García et al. (2013) [27] found that ENNB increased the percentage of HepG2 cells arrested at G0/G1 at 48 h suggesting an anti-proliferative activity of ENNB. In Caco-2 cells, 2-day to 3-day treatment of ENNB (3 μM) caused the arrest of the cell cycle in the G2/M phase [28,29]. The present study is the first to show potent inhibitory effects of ENNA on the proliferation of ovarian GC. Previous studies evaluating the effects of Fusarium mycotoxins on granulosa cell proliferation show that fumonisin B1 and beauvericin reduce cell growth after 24 h and 48 h of treatment [23,24] but the mechanism of inhibition was not evaluated. A recent study has reported that beauvericin reduces the mRNA abundance of ubiquitin-like with plant homeodomain and really interesting new gene finger domains 1 (UHRF1), a multi-domain nuclear protein that plays an important role in DNA repair and DNA replication controlling the onset of S phase [30], but whether ENNA affects GC proliferation via UHRF1 will require further study. Further work will also be required to determine the stage of the cell cycle that is disrupted by ENNA as well as evaluate if ENNA activates programmed cell death. The ENNA IC_50_ identified for GC proliferation in the present study (i.e., 2 µM) is similar to toxicity assay IC_50_ reported for other cell types including J774 macrophages [31], CHO-K1 cells [32], and neuroblastoma cells [33].

Other possible modes of action of ENNA can be inferred from studies of Hoornstra and co-workers [34] who found that 500 ng/mL (0.7 µM) ENNA, ENNA1, ENNB, and ENNB1 inhibited sperm motility by depolarizing the mitochondria and hyperpolarizing the plasma membrane of sperm cells [34]. Also, it was observed that sperm cells exposed to 0.6 µg/mL (1 µM) ENNB in media with 4 mM (physiological) or 1 mM (low) concentration of K+, had depolarization of mitochondrial membrane potential [35]. Mitochondria play a key role in GC steroidogenesis [36,37,38], but whether depolarization of mitochondria is involved in how ENNA affects steroid production in GC will require further study. Perhaps this is why steroid production of large-follicle GC was more sensitive to ENNA than cell proliferation in the present study. In Leydig cells, 0.01–10 µM ENNB did not affect the viability of cells, whereas 100 µM ENNB caused a 21% loss of cell viability in LH-stimulated Leydig cells [39]. In LH-stimulated Leydig cells, 0.01 and 10 µM ENNB had no effect on testosterone production whereas 100 µM ENNB reduced estradiol and testosterone production suggesting that at high (100 µM) doses, ENNB inhibits Leydig cell viability and steroid production. Kalayou et al. (2015) [39] also reported that 10 µM ENNB inhibited progesterone and cortisol production by human adrenocortical carcinoma cells. In the present study, much lower doses of ENNA (i.e., 0.3–5 µM) inhibited steroid production by GC. Thus, ENNs significantly reduced steroid production in testicular, adrenal, and ovarian cells. Based on concentrations of ENNA reported in livestock feed [40,41,42,43], maximal concentrations of ENNA achieved in the blood of cattle would be estimated to range between 0.4 nM and 2.8 µM. Because ENNs co-occur with other mycotoxins in food and feed, additional in vitro studies evaluating ENNs and other emerging mycotoxin combinations are needed [8,16,36].

## 4. Conclusions

In conclusion, these results demonstrate that ENNA inhibits bovine granulosa cell proliferation and steroidogenesis in a dose-dependent manner suggesting its potential to impair reproductive function in cattle and act as a natural endocrine disruptor. Additional research is required to ascertain the intracellular mechanism(s) by which GC proliferation and steroidogenesis are inhibited by ENNA. Moreover, it is clear that more data on ENNs, alone or in a mixture, are urgently required for a correct risk assessment, and a future legislative approach.

## 5. Materials and Methods

### 5.1. Tissues, Hormones, and Reagents

There were no live animals used in this study, so no ethical approval was required. Bovine ovaries were collected at an abattoir where humane slaughter practices were followed, according to USDA guidelines. The hormones and reagents used in cell culture were: ovine follicle-stimulating hormone (FSH; NIDDK-oFSH-20; activity: 175 × NIH-FSH-S1 U/mg) from the National Hormone and Pituitary Program (Torrance, CA, USA), recombinant human IGF1 from R&D Systems (Minneapolis, MN, USA); testosterone from Steraloids (Wilton, NH, USA); and fetal calf serum (FCS) from Atlanta Biologicals (Atlanta, GA, USA); ENNA, Dulbecco’s modified Eagle’s medium (DMEM), Ham’s nutrient mixture F-12 (F12), collagenase, DNase, gentamycin, glutamine, and sodium bicarbonate from Sigma-Aldrich, Inc. (St. Louis, MO, USA).

### 5.2. Cell Culture

Ovaries from non-pregnant beef cows were collected from a local abattoir and based on surface diameter, GC was collected and prepared from small (1 to 5 mm) and large (8 to 22 mm) follicles as previously described [24,44]. The viability of GC from small and large follicles was determined by the trypan blue exclusion method and averaged 58 ± 7% and 64 ± 6%, respectively, and is similar to viabilities reported in previous studies [45,46,47,48].

Viable cells (2 × 10^5^ cells in 28–75 μL of medium) were plated on 24-well Falcon multiwell plates (Becton Dickinson, Lincoln Park, NJ, USA) in basal medium (1 mL) containing 10% FCS (v/v) and cultured at 38.5 °C in 10% FCS for the first 48 h as previously described [23,24,45,49]. The various treatments were applied in serum-free medium containing testosterone (500 ng/mL, as an estrogen precursor) for 48 h with a medium change at 24 h. The medium was collected for steroid radioimmunoassay (RIA) and cells were collected for cell counting (see Section 5.3). The concentrations of FSH and IGF1 were selected based on previous studies [24,45,50]. Because steroid production in this culture system is maximized with a combined treatment of FSH and IGF1 and only weakly responsive to either FSH or IGF1 alone [24,45], FSH was used in combination with IGF1 for all experiments.

Experiment 1 evaluated the dose-response effect of ENNA on hormone-induced proliferation and steroidogenesis of bovine GC from large follicles. The GC from large follicles was collected, cultured for 48 h in 10% FCS and then cultured for an additional 48 h in the presence of FSH (30 ng/mL), IGF1 (30 ng/mL), and testosterone (500 ng/mL, as an estrogen precursor) with various doses of 0, 0.3, 1 and 3 µM ENNA. The medium was changed every 24 h. Cells were enumerated on day 2 and the medium was collected on days 1 and 2 for RIA to measure estradiol and progesterone concentrations (see Section 5.3). Doses of ENNA were selected based on previous in vitro studies [21,22,34,51].

Experiment 2 evaluated the dose-response effect of ENNA on hormone-induced cell numbers and steroid production of small-follicle GC. Cells were cultured for 48 h in 10% FCS and then cultured for an additional 48 h in the presence of FSH (30 ng/mL), IGF1 (30 ng/mL), and testosterone (500 ng/mL) with or without various doses of ENNA (0, 1, 3 and 5 µM). Cells were counted on day 2 and the medium was collected on days 1 and 2 for RIA to measure estradiol and progesterone concentrations (see Section 5.3). Doses of ENNA were selected based on previous in vitro studies [21,22,34,51]. The 24 h and 48 h treatment durations in Experiments 1 and 2 were selected based on our previous studies with other mycotoxins [23,24].

### 5.3. Determination of Steroid Concentrations and Cell Numbers

The medium was collected from individual wells and frozen at −20 °C for subsequent determination of concentrations of estradiol and progesterone via RIA as previously described [24,48]. A 24 h period of testosterone exposure to GC allows for a direct measure of functional aromatase activity [49]. The intra-assay coefficients of variation averaged 5.8% and 9.3% for the progesterone and estradiol RIA, respectively. The numbers of cells in the same wells in that medium was collected were determined as previously described [24,48,52] using a Coulter counter (model Z2; Beckman Coulter, Inc., Miami, FL, USA). The intra-assay coefficients of variation averaged 2.0 ± 0.8%.

### 5.4. Statistical Analysis

Data are presented as the least squares means (± SEM) of measurements from three pools of cells (i.e., biological replicates) with each pool of cells collected from at least 10 individual animals for small-follicle GC and at least 5 individual animals for large-follicle GC, and each replicated experiment (i.e., a pool of cells) had three technical replicates of cells per treatment. Treatment effects and interactions were determined via ANOVA using the general linear models procedure of SAS for Windows (ver. 9.4, SAS Institute Inc., Cary, NC, USA). Exp. 1 and 2 were analyzed as 2 × 4 factorial ANOVA with day and ENNA dose as the main effects and their interactions. Specific differences in cell numbers and steroid production were determined using Fisher’s protected least significant difference procedure [53]. Significance was declared at *p* < 0.05. 

## Data Availability

The data sets generated during the current study are available from the authors upon request.
